# Risk factors of the post-reperfusion syndrome during orthotopic liver transplantation: a clinical observational study

**DOI:** 10.1186/s12871-022-01635-3

**Published:** 2022-04-02

**Authors:** Mohammad Ali Sahmeddini, Samaneh Ghazanfar Tehran, Mohammad Bagher Khosravi, Mohammad Hossein Eghbal, Naeimehossadat Asmarian, Fatemeh Khalili, Pooya Vatankhah, Somayeh Izadi

**Affiliations:** 1 Anesthesiology and Critical Care Research Center, Shiraz University of Medical Sciences, Shiraz, Iran; 2 Anesthesiology Research Center, Department of Anesthesiology, Alzahra Hospital, Guilan University of Medical Sciences, Rasht, Iran; 3 Shiraz Transplant Center, Abu-Alisina Hospital, Shiraz University of Medical Sciences, Shiraz, Iran

**Keywords:** Liver, Liver Diseases, Transplantation

## Abstract

**Background:**

Post reperfusion syndrome (PRS) is a relatively common and life-threatening complication during orthotopic liver transplantation (OLT). It is associated with poor patient and transplanted liver outcomes.

**Objective:**

This study aimed to compare the risk factors of PRS during OLT.

**Design:**

Clinical-epidemiological observational retrospective study.

**Setting:**

We gathered the records of patients who underwent OLT in 3 years, from May 22, 2016, to May 22, 2019, in Namazi and Bu-Ali Sina organ transplantation hospitals.

**Patients:**

In this study, we assessed 1182 patients who underwent OLT. Patients were divided into two groups based on the presence or absence of PRS.

**Main outcome measures:**

Diagnosing the predictors of PRS was the primary outcome of this study.

**Results:**

Results showed that age > 60 years, Child-Pugh scores C, higher Model End Stage liver disease score, and preoperative sodium < 130 mmol/l (parameters of the liver recipient), increase in cold ischemic time (the donors’ parameters), and the classical technique (the surgical parameters) were the strong predictors of PRS.

**Conclusions:**

The results indicated that underlying liver disease was not the predictor of PRS in the presence of other risk factors; therefore, clinicians have to consider these risk factors in patients undergoing OLT.

**Supplementary Information:**

The online version contains supplementary material available at 10.1186/s12871-022-01635-3.

## Key points

- Liver transplantation is a life-saving procedure and the treatment of choice for severe chronic liver disease and acute liver failure patients.
- Post reperfusion syndrome is a relatively common but life-threatening complication during liver transplantation.
- This study showed that 33% of patients who underwent liver transplantation at this center had PRS.
- Age > 60 years, CTP class C, higher MELD score, preoperative sodium < 130 mmol/l, CIT, and classical technique were PRS predictors.

## Introduction

Orthotopic Liver transplantation (OLT) is a life-saving procedure and the treatment of choice for severe chronic liver disease and acute liver failure [1, 2]. Post reperfusion syndrome (PRS) is a relatively common but life-threatening complication during OLT with a prevalence of 3.6–81% [3–6]. The wide range prevalence of PRS in the previous studies occurs due to the differences in the description of PRS, treatment strategies, and surgical techniques [7]. PRS is the acute hemodynamic disturbance and decreased mean arterial pressure of more than 30% of baseline values. It occurs within the first 5 min after reperfusion of the donor’s liver with the recipient’s blood during the transition from the anhepathic phase to the neohepathic phase, which lasts for at least 1 min [4, 8, 9]. It is associated with poor patient and transplanted liver outcomes [3, 10, 11]. Although the definitive mechanism of this syndrome is complex and not fully understood [7, 9, 12–14], it is attributed to the release of oxygen free radicals, endotoxin, inflammatory cytokines such as tumor necrosis factor-alpha (TNF-α), interleukin-1 (IL-1), IL-2, vasoactive, hyperkalemic, cold, and acidotic substances of maintenance solution, donor’s liver, and recipient ischemic intestine [6, 9, 14, 15].

OLT is major surgery with a high occurrence of fluid shift and is often performed in patients with hemodynamic changes associated with the pathology of the underlying liver disease. On the other hand, reperfusion syndrome is a severe complication during surgery following portal vein declamp. Despite the improvement of surgical and anesthesia techniques, it is associated with severe metabolic and cardiovascular disorders that can affect mortality and morbidity of patients [6, 15]. Postoperative acute kidney injury (AKI), early allograft dysfunction, reduced graft, and the patient’s survival rate are related to PRS [16, 17]. Therefore, understanding the risk factors associated with this syndrome is very beneficial. Considering the importance of this subject, investigators identified the risk factors related to PRS. However, so far, few studies have been conducted to investigate the relationship between the type of underlying liver disease leading to OLT with the incidence of PRS and the prevalence of this complication separately based on the liver disease [18]. Since many patients with conditions such as viral hepatitis, cancers, and biliary, autoimmune, metabolic, and cryptogenic diseases undergo OLT, we aimed to investigate the prevalence of PRS separately based on liver disease. We assessed the relationship between liver disease and other risk factors for PRS in patients who underwent OLT.

## Materials and methods

This clinical-epidemiological observational retrospective study extracted data from May 22, 2019, to November 22, 2019. We gathered the records of patients who underwent OLT in 3 years, from May 22, 2016, to May 22, 2019, in Namazi and Bu-Ali Sina organ transplantation hospitals. The Ethical Committee of the Vice-chancellor of Research (Chairperson: Dr. Mohammad Javad Ashraf) at Shiraz University of Medical Sciences approved this study (Number: IR.SUMS.MED.REC.1398.170, Date: May 22, 2019). We conducted this study to determine the predictors of PRS, including the type of underlying liver disease leading to transplantation and other risk factors.

### Participants

Inclusion criteria included adult patients (age ≥ 18 years) with end-stage liver disease who underwent deceased-donor OLT.

Exclusion criteria included OLT from a living donor, split liver graft, simultaneous liver and kidney transplantation, patients with acute liver failure, incomplete information recorded before and during surgery, and the occurrence of intraoperative severe adverse events before the reperfusion phase.

### Anesthesia protocol

Upon admission to the operating room and after appropriate venous line placement for all patients, we performed non-invasive blood pressure monitoring, electrocardiogram, and pulse oximetry. Besides, we placed the arterial line for invasive pressure monitoring, the central venous line under ultrasonography for central pressure, temperature probe, and capnography immediately after induction of anesthesia. PICCO monitoring and, if needed, TEE was used to improve patient management in patients with high MELD (model for end-stage liver disease), heart problems, or aortopulmonary shunt.

We performed induction of anesthesia with the injection of 0.02 mg. Kg-1 midazolam, 2 μg. Kg-1 fentanyl, 3-5 mg. Kg-1 sodium thiopental and 0.2 mg. Kg − 1 pancuronium. The anesthesia maintenance was done using isoflurane with MAC of less than 1% in combination with oxygen/air.

After intubation, we provided mechanical ventilation with a tidal volume of 8-10 ml / kg, respiratory rate of 10–16 breaths/min, and 5cmh2o PEEP to maintain end-tidal CO2 partial pressure (PETCO2) of 35–45 mmHg for the patient. Albumin and normal saline were used for fluid therapy based on hemodynamic parameters and central venous pressure. Patients were monitored to maintain systolic blood pressure and heart rate at 20% of baseline during surgery. We performed necessary interventions in case of hemodynamic changes using anesthetics, cardiovascular drugs, and fluids.

### Surgical technique

Based on the patient’s condition and the surgeon’s decision, we used piggyback or classical techniques during surgery for OLT. We did not use venovenous bypass during the surgery. Before reperfusion, the graft was rinsed with 1 ml / kg normal saline through the portal vein.

### Data gathering

#### Donors’ data

We extracted the following donor’s data from the records:

Age, sex, length of stay in the intensive care unit (ICU), the blood concentration of alanine transferase (ALT), aspartate transferase (AST), alkaline phosphatase, bilirubin, sodium, and potassium levels, and preoperative international normalized ratio (INR), as well as warm ischemia time (WIT) graft, and cold ischemia time (CIT) graft.

#### Recipient’s data

We collected the recipient’s data from patients’ records. It included age, sex, weight, type of underlying liver disease leading to transplantation, preoperative sodium level, MELD (a scoring system based on laboratory tests including creatinine, bilirubin, INR, and serum sodium levels ranging from 6 to 40), [19], and Child-Pugh scores (CTP) which is a scoring system based on five parameters: total bilirubin level, albumin, prothrombin time or INR, ascites severity, and degree of encephalopathy, which receives 1 to 3 points per parameter. According to the acquired score, patients were placed in 3 classes, including A (score 5–6), class B (score 7–9), and class C (score 10 to 15). A and C classes represented mild and severe diseases, respectively [19]. Also, we assessed intraoperative hemodynamic parameters such as heart rate, systolic and diastolic blood pressures, mean arterial pressure, major cardiovascular events nd the amount of consumed inotrope after reperfusion.

Following adverse events occurring in ICU were evaluated as well: primary graft nonfunction (PGNF: retransplantation or death within 7 days), need for renal replacement therapy (RRT), major cardiovascular events (cardiac arrest, myocardial infarction, or significant arrhythmia), need for surgical revision (portal vein thrombosis, hepatic artery thrombosis, bleeding, biliary duct complications, intraabdominal abscess/peritonitis), central nervous system events (delirium, depression, and seizure), length of stay in ICU and hospital, and finally the outcome of patients before discharge and within 2 years of follow-up after discharge.

#### PRS

PRS was noted by the decreased mean arterial pressure of more than 30% of baseline values within the first 5 min after PRS of the donor’s liver with the recipient’s blood during the transition from the anhepathic phase to neohepathic phase, which lasts for at least 1 min. We used norepinephrine as a bolus and infusion to maintain mean arterial pressure (MAP) for this complication. In case of hypotension along with severe bradycardia, we used epinephrine bolus and infusion. Finally, we used vasopressin infusion in case of the need for a high dose of norepinephrine or its ineffectiveness.

After extracting information from the records, based on the occurrence or non-occurrence of this complication, patients were compared in groups 1 (PRS +) and 2 (PRS -).

### Statistical analysis

Data were analyzed in IBM SPSS Statistics for Windows, version 19 (IBM Corp., Armonk, N.Y., USA). The continuous variables were reported by median (IQR) and tested by the Mann-Whitney U test. The categorical variables were reported by number (percent) and tested by Chi-square or Fisher exact. Univariate and multivariable logistic regression models were used to determine risk factors for predicting PRS. In logistic regression models, we have to consider one category of categorical variables as the reference. In this study, we chose the variable with the highest risk of RPS as the reference. *P*-value< 0.05 indicated statistical significance.

## Results

**Fig 1:**
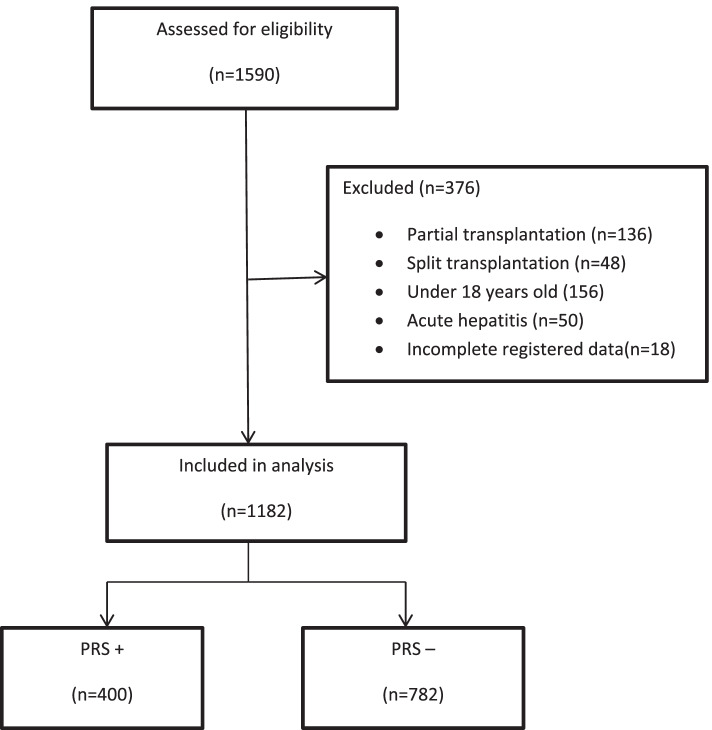
Flow chart of patients

A total of 1590 patients underwent OLT within 3 years of the study (Fig. 1). Of these, 376 patients were excluded from the study, including 136 patients due to partial transplantation from a living donor, 48 patients due to split transplantation, 156 patients under 14 years of age, 50 patients due to acute hepatitis, and 18 patients due to incomplete registered information. Finally, we assessed 1182 patients who underwent OLT.

### Baseline characteristics of studied patients

Demographic characteristics of liver recipients, including age, sex, weight, distribution of underlying liver disease, MELD Score, Child Score, preoperative sodium levels, are shown in Table 1. In terms of age distribution, most patients are in the age range of 40–60 years, and the lowest patients were in the under-20 years’ category. Results showed that 65% of patients were men. In terms of CTP score, most patients were in class C and the least in class A. The mean MELD score of the patients was 20 (20.01 ± 8.15). The most common causes of OLT within 3 years of study were a biliary disease, viral hepatitis, metabolic, cryptogenic, Autoimmune hepatitis (AIH) disorders, and cancers, respectively (Table 1).

**Table 1:** Clinical characteristics of liver transplant recipients

Recipients characteristics	PRS(−) *N* = 782	PRS(+) *N* = 400	*P*-value
Age			
< 20	26 (3.3)	4 (1)	< 0.001
20–40	279 (35.7)	98 (24.5)	
40–60	390 (49.9)	229 (57.3)	
> 60	87 (11.1)	69 (17.3)	
Sex, female	271 (34.7)	142 (35.5)	0.773
Weight	70 (60–80)	70 (60–80)	0.227
MELD Score for chronic liver disease	17 (13–21)	22 (18–29)	< 0.001
CTP Score			
Score A	219 (28)	17 (4.3)	< 0.001
Score B	351 (44.9)	117 (29.3)	
Score C	212 (27.1)	266 (66.5)	
Recipient Pre-operative Na			
Na < 130	45 (5.8)	71 (17.8)	< 0.001
Na ≥ 130	735 (94.2)	328 (82.2)	
Chronic Liver Disease			
Viral Hepatitis	149 (19.1)	110 (27.5)	< 0.001
Metabolic disease	127 (16.2)	77 (19.3)	
Biliary disease	235 (30.1)	72 (18)	
AIH	100 (12.8)	43 (10.8)	
Hepatic cancers	56 (7.2)	27 (6.8)	
cryptogenic	115 (14.7)	71 (17.8)	

*PRS* Post reperfusion syndrome, *MELD* Model for end-stage liver disease, *CTP* Child-Turcotte-Pugh

Table 2 shows the demographic characteristics, laboratory tests, and data of WIT and CIT of liver organ donors. The majority of donors were men. Mean CIT and WIT were 445(445.25 ± 157.43) and 36 (36.64 ± 7.61), respectively. Serum levels of sodium, potassium, AST, ALT, ALKP, bilirubin, and INR were similar between the two groups (Table 2).

**Table 2:** Clinical characteristics of liver organ donor

Donor characteristics	PRS(−) *N* = 782	PRS(+) *N* = 400	*P*-value
Age			
≤ 60	710 (90.8)	341 (85.3)	0.004
> 60	72 (9.2)	59 (14.8)	
Sex, female	258 (33)	140 (35)	0.49
ICU state (day)	4 (3–7)	4 (3–7)	0.222
Na (meq)	147 (141–153)	148 (141–154)	0.034
AST	51 (32–96)	46 (29–86)	0.056
ALT	40 (22–79)	40 (22–85)	0.973
ALKP	186 (145–256)	193 (150–254)	0.248
Bilirubin	0.8 (0.5–1)	0.8 (0.5–1)	0.675
INR	1.3 (1.2–1.5)	1.3 (1.1–1.5)	0.266
CIT (min)	480 (360–540)	480 (420–570)	< 0.001
WIT (min)	35 (30–40)	35 (30–40)	0.132
Surgery technique, piggy back	607 (77.6)	268 (67)	< 0.001

*PRS* Post reperfusion syndrome, *ICU* Intensive Care Unit, *AST* Aspartate transferase, *ALT* Alanine transferase, *ALKP* Alkaline phosphatase, *INR* International normalized ratio, *CIT* Cold ischemia time, *WIT* Warm ischemia time

### The PRS

Out of 1182 patients, 400 patients (33.8%) had PRS. There was a significant difference between groups regarding underlying disease leading to transplantation. We found the highest incidence of PRS in the viral hepatitis group and the lowest in the biliary disease group (42.5% vs. 23.5%, *P* < 0.001). In terms of age distribution, there was a significant difference between groups. The highest incidence of PRS was in patients aged more than 60 years, and the lowest incidence was in patients under 20 years (44.2% vs. 13.3%, *P* < 0.001). But in terms of weight and sex, there was no significant difference. Regarding disease severity distribution between the two groups, a higher incidence of PRS was noted in patients with higher MELD and CTP scores. The highest incidence of PRS occurred in patients with CTP C compared to A (55.6% vs. 7.2%, *P* < 0.001). Also, assessing the effect of preoperative sodium on PRS showed that patients with sodium less than 130 compared to sodium more than 130 had a higher incidence of this complication (61.2% vs. 30.9%, *P* < 0.001) (Table 1).

Also, transplant recipients from liver donors older than 60 years showed a higher percentage of reperfusion than donors less than 60 years old (45% VS. 32.4%, *P* = 0.003). However, there was no significant difference between the two groups regarding sex and test levels of K, AST, ALT, ALKP, Bilirubin, and INR before donation (*P* > 0.05). Only in donors with higher sodium levels, more PRS was observed (*P* = 0.03). Donor organs also had a longer CIT for patients with PRS (*P* = < 0.001). But in terms of WIT, no statistically significant difference was observed between the groups (*P* = 0.132) (Table 2).

### Predictors of the PRS

To determine PRS predictors, we used binary logistic regression analysis (Table 3). Based on the results obtained from the parameters of the liver recipient, the following parameters were strong predictors of PRS. Age > 60 years (The odds ratio of PRS in age > 60 were respectively 42, 59, and 78% more than 40–60, 20–40, and 20 years). CTP class C (The odds ratio of PRS in class C was about 84 and 56% more than A and B). Higher MELD score (The increase in MELD score per unit increased the odds ratio of developing PRS by 6%). Preoperative sodium < 130 mmol/l (The odds ratio of PRS in sodium < 130 was about 45% more than sodium > 130).

**Table 3:** Adjusted odds ratios derived from logistic regression analysis for patients with PRS

	Un-adjusted model			Adjusted model		
	OR	95% CI	***P***-value	OR	95% CI	***P***-value
Age (year)						
> 60	Reference category of the variable					
< 20	0.19	0.06–0.58	0.003	0.22	0.07–0.71	0.011
20–40	0.44	0.30–0.65	< 0.001	0.41	0.25–0.68	< 0.001
40–60	0.74	0.52–1.06	0.098	0.58	0.38–0.88	0.011
Chronic Liver Disease						
Viral hepatitis	Reference category of the variable					
Metabolic Disease	0.82	0.56–1.19	0.304	0.78	0.51–1.20	0.259
Biliary Disease	0.41	0.29–0.60	< 0.001	0.78	0.51–1.21	0.274
AIH	0.58	0.38–0.90	0.015	0.63	0.38–1.05	0.077
Hepatic Cancers	0.65	0.39–1.10	0.109	0.90	0.46–1.74	0.751
Cryptogenic	0.84	0.57–1.23	0.363	0.77	0.49–1.19	0.240
CTP Score						
Child Score C	Reference category of the variable					
Child Score A	0.06	0.04–0.10	< 0.001	0.16	0.09–0.30	< 0.001
Child Score B	0.27	0.20–0.35	< 0.001	0.44	0.32–0.62	< 0.001
MELD Score	1.12	1.10–1.14	< 0.001	1.06	1.04–1.09	< 0.001
Recipient Pre-operative Na						
< 130	Reference category of the variable					
≥ 130	0.28	0.19–0.42	< 0.001	0.55	0.36–0.86	0.008
Donor age						
> 60	Reference category of the variable					
≤ 60	0.59	0.41–0.85	0.004	0.91	0.59–1.40	0.662
Donor Na	1.01	1.00–1.03	0.068	1.01	0.999–1.03	0.067
CIT (min)	1.001	1.001–1.002	0.002	1.002	1.001–1.003	< 0.001
Surgery technique						
Classical	Reference category of the variable					
piggy back	0.58	0.45–0.76	< 0.001	0.59	0.43–0.82	0.001

(−):Reference category of the variable, *AIH* Autoimmune hepatitis, *MELD* Model for end-stage liver disease, *CTP* Child-Turcotte-Pugh, *CIT* Cold ischemia time

Besides, among the donors’ parameters, only an increase in CIT was the strong predictor (per each minute increase in CIT, the odds ratio of PRS increased 0.2%). Among the surgical parameters, the classical technique (the odds ratio of PRS in classical technique was 41% more than the piggyback technique) was the strong predictor of PRS compared to the type of underlying liver disease, donors’ age, and sodium level. In the case of underlying liver disease and through univariate analysis, the odds ratio of PRS in biliary and autoimmune diseases was significantly lower than in viral hepatitis. However, in multivariate analysis, this factor was not indicated as a predictor of PRS (Table 3).

### PRS and post-liver transplantation adverse events and outcomes

Our study showed that the rate of renal dysfunction requiring RRT in LT recipients with PRS was significantly higher than the other group (18% vs 3.2%; *P* < 0.001). Patients with postoperative PRS also experienced more delirium (10.5 vs 5.1%; *P* < 0.001) and major cardiovascular event (11.5% vs 2.6%; *P* < 0.001). The need for postoperative surgical revision was higher in the PRS group than in the other group, of which bleeding was the most common cause of surgical revision. The length of hospitalization in the ICU as well as the hospital in the group with PRS was significantly longer than the other group (*P* = 0.14, *P* = 0.044, respectively). There was a significant difference between the groups in the rate and causes of death in the postoperative period. The mortality rate in the PRS group was higher than the other group (27.25% vs 5.11%; *P* < 0.001) and PGNF, multiple organ failure (MOF) / sepsis and bleeding were the most common causes of death in the initial period after LT, respectively. Also in the two-year follow-up after discharge, it was found that the mortality rate in the PRS group was significantly higher than other group (13.7% vs 8.2%; *p* = 0.007, respectively) and MOF / sepsis and chronic rejection were the most common causes of death in both groups, respectively (Table 4).

**Table 4:** Intra operative cardiovascular events and post-operative outcomes

Variable	PRS – (***N*** = 782)	PRS + (***N*** = 400)	***P*** Value
**Intra operative cardiovascular events(n)**			
Cardiac arrest	0 (0)	5 (1.25)	0.004
Significant Arrhythmia	7 (0.89)	47 (11.7)	< 0.001
**Short-term outcomes:**			
**RRT due to Renal dysfunction (n)**	25 (3.2)	72 (18)	< 0.001
**Major Cardiovascular events (n)**	20 (2.6)	46 (11.5)	< 0.001
**Surgical revision due to:**			
H.A Thrombosis(n)	44 (5.6)	19 (4.7)	0.526
PV Thrombosis(n)	9 (1.1)	8 (2)	0.246
Bleeding(n)	52 (6.6)	100 (25)	< 0.001
Biliary duct complication(n)	8 (1)	6 (1.5)	0.473
Intra-abdominal Abscess/ peritonitis(n)	2 (0.25)	2 (0.5)	0.494
**CNS events**			
Delirium(n)	40 (5.1)	42 (10.5)	0.001
Seizure(n)	40 (5.1)	27 (6.75)	0.250
Depression(n)	20 (2.6)	8 (2)	0.551
**ICU Length (day)**	8 (5–10)	9 (5–14)	0.014
**Hospital Length (day)**	12 (9–15)	14 (9–22)	0.044
**Post-operative Deaths (n)**			
Due to PGNF (n)	8 (1)	37 (9)	< 0.001
Due to MOF/sepsis (n)	29 (3.7)	63 (15.7)	< 0.001
Due to bleeding (n)	0 (0)	5 (1.25)	0.004
Due to cardiovascular events (n)	3 (0.38)	4 (1)	0.235
**Long-terms outcomes**			
**Death within 2 years of follow up**	**(*N*** **= 742)**	**(*N*** **= 291)**	
Due to MOF/sepsis (n)	21 (2.8)	16 (5.5)	0.038
Due to cardiovascular events (n)	7 (0.94)	5 (1.7)	0.296
Due to respiratory problems (n)	11 (1.5)	5 (1.7)	0.782
Due to chronic rejection(n)	12 (1.6)	9 (3.1)	0.131
Due to carcinoma (n)	10 (1.3)	5 (1.7)	0.654

*RRT* Renal replacement therapy, *CNS* Central nervous system, *HA Thrombosis* Hepatic artery thrombosis, *PV Thrombosis* Portal vein thrombosis, *ICU* Intensive care unit, *PGNF* Primary graft nonfunction, *MOF* Multiple organ failure

## Discussion

This study showed that 33.8% of patients at this center had PRS. Results showed the following parameters as the strong predictors of PRS: age > 60 years, Child-Pugh scores C, higher MELD score, and preoperative sodium < 130 mmol/l (parameters of the liver recipient), increase in cold ischemic time (the donors’ parameters), and the classical technique (the surgical parameters).

The incidence of PRS varies from 3.6–81% among studies [3, 5]. The most important reason for this wide range seems to be the significant differences in PRS descriptions. Also, other factors such as patient population and surgical technique were significantly different in previous studies, which can affect the PRS rate and explain the high difference in the incidence of this complication in studies. The incidence of PRS reported in most studies was 30% [20–22]. In the present study, the incidence of this complication was about 33%, which was consistent with other studies. In Fakhar et al.’s study, this complication rate was 12% due to shorter ischemia times and better donor conditions [5]. But in the survey by Kork et al., the incidence of PRS was 53%, attributed to the use of more comprehensive criteria to diagnose this complication [3].

Assessing the distribution of liver disease leading to OLT, our results showed that biliary disease, viral hepatitis, and metabolic diseases were the most common causes of OLT from 2016 to 2019. In a previous Iranian study examining the status of OLT before 2016, the most common indications of OLT were cirrhosis caused by viral hepatitis, cryptogenic, and biliary disease [23]. The difference in the order at different periods of investigations may be attributed to the following parameters: increased biliary liver disease, mainly primary sclerosing cholangitis (PSC) due to the inflammatory bowel disease epidemic [1, 24], increased obesity and metabolic syndrome, the most important predictors of non-alcoholic fatty liver disease/steatohepatitis (NAFLD / NASH) [25], and decreased new viral infections due to vaccination for hepatitis B as well as the new generation of drugs for hepatitis C [1].

Although the exact pathophysiological mechanism of PRS is not fully understood, several studies have attempted to provide a list of possible risk factors for PRS to improve prevention and treatment strategies. Based on the results of studies, these risk factors were divided into three categories: donor/organ-related, recipient-related, and procedure-related [6].

Most of the risk factors were related to liver graft recipients [6]. Finding a relation between hyponatremia and PRS was one of the most important findings of this study related to the recipient. Hyponatremia is the most common electrolyte abnormality in patients with advanced cirrhosis and predicts poor prognosis. Hyponatremia in cirrhosis is defined as a sodium concentration < 130 mmol / l and has a prevalence rate of 22%. The pathophysiology of hyponatremia in these patients is complex and multifactorial. Although it is predominantly hypervolemic or dilutional, it can be hypovolemic due to excessive diuretic or gastrointestinal losses such as diarrhea in only 10% [26, 27]. No study investigated the association between hyponatremia and PRS. This study showed that the recipient’s hyponatremia in the case of sodium less than 130 was a predictor of PRS; therefore, sodium less than 130 caused a 45% increase in the incidence of PRS.

In terms of the incidence of PRS based on the underlying liver disease leading to OLT, so far, the studies compared the incidence of PRS between the two groups of patients with acute and chronic hepatitis. To the best of our knowledge, few studies investigated the incidence of this complication among chronic liver diseases. The univariate analysis on the incidence of PRS based on chronic underlying liver disease showed that the highest incidence of PRS occurred in patients with viral hepatitis (42%) and the lowest in patients with biliary (23%) and autoimmune diseases (30%). We observed a significant difference between the groups (*p*-value < 0.05). However, when we assessed this parameter in the multivariate analysis and other risk factors for PRS, no significant difference was observed between the underlying liver diseases in terms of PRS, so the underlying liver disease was not recognized as the PRS predictor. This result indicated the greater importance of other risk factors for PRS than the recipient’s underlying liver disease. The cause of higher incidence of PRS in patients with viral hepatitis than biliary and autoimmune diseases can be attributed to older age and higher severity of underlying diseases such as higher MELD and CTP scores in these patients. In an abstract examining the risk factors of PRS, there was no significant correlation between PRS and the etiology of liver disease [18].

In terms of age, studies have shown that the recipients’ age > 60 years is a risk factor for PRS [22]. In this study, in line with previous studies, age > 60 years was one of the predictors of this complication. So that the odds ratio of developing PRS over the age of 60 was 78% higher than under the age of 20. However, in the study of Kork et al., there was no difference between the two groups regarding age [4].

The most important risk factors related to donor and liver graft resulting from studies were the age of the donor over 60 years, long CIT [6, 22], and Donor Risk Index [6]. This study showed that among the risk factors for donor-related PRS, only long-term CIT was the predictor for this complication. CIT causes liver damage with primary necrosis of sinusoidal endothelial cells and delayed hepatocyte apoptosis. This study also showed that the classical technique was associated with a higher incidence of PRS than the piggyback technique. The odds ratio of PRS with the classical technique was about 41% higher than the piggyback technique. The result of this study was consistent with previous studies [6, 7].

One notable finding of our study was the association between the occurrence of PRS with postoperative poor liver graft and patient’s outcome. The incidence of PGNF and mortality in the PRS group was significantly higher than the other group. In the study of Siniscalchi et al., PRS was identified as an independent risk factor for PGNF and mortality [16]. However, in the study of Kork et al., which examined the effect of PRS on graft function and mortality, despite the higher PGNF in the group with PRS, no significant relationship was observed between the two groups [4]. It can be attributed to the small sample size in their study in contrast to ours and Siniscalchi et.al. One of the important findings in our study was that more renal dysfunction required RRT in the PRS group. In a meta-analysis performed by Zhou et al. PRS was identified as one of the AKI risk factors after LT [28]. Hemodynamic instability during PRS has a major effets on decreased renal blood flow and renal tissue hypoxia, which can play an important role in the development of AKI after LT, on the other hand, PRS not only releases cold and acidotic components from the graft but also releases pro-inflammatory cytokines that trigger the inflammatory response and cause renal tubular injury [28] .In terms of the need for surgical revision, in the initial period after transplantation, although in our study, bleeding was the most common reason for the need for reoperation in both groups, there was significant difference between them. So that a higher percentage of patients in the PRS group required surgical revision due to bleeding. Postoperative bleeding is the most common serious complication after LT that is life-threatening and requires surgery to control bleeding or hematoma drainage. Early graft dysfunction or PNF, liver surface laceration, anastomotic leak, and hepatic artery stenosis and thrombosis are the leading causes of postoperative bleeding [29]. More bleeding in the PRS group can be attributed to the greater incidence of graft dysfunction in this study. Consistent with the previous study [4], we also showed that PRS is associated with a higher frequency of delirium and major cardiac events after transplantation, followed by more extended hospital stays in the ICU and hospital.

So far, many studies have been performed to diagnose PRS risk factors. Still, according to this search, few studies have been conducted to investigate the incidence of PRS based on underlying liver disease leading to OLT. On the other hand, the sample size of this study was large, and so far, no study with this size has been conducted to evaluate the incidence of PRS, which can be the strengths of this study. As we performed a retrospective single-center study, further prospective mutlicenter studies can berecommened.

## Conclusion

The study showed that among the PRS risk factors related to the organ recipient, age > 60 years, CTP class C, higher MELD score, preoperative sodium < 130 mmol/l, and among the PRS risk factors related to the organ donor, only CIT and among PRS risk factors related to surgery technique, classical technique were PRS predictors. Despite the significant differences between liver diseases leading to OLT in PRS, it was not the OLT predictor. This result indicated the greater importance of other risk factors for PRS than the recipients’ underlying liver disease. Therefore, clinicians have to consider these risk factors in patients undergoing OLT.

## Supplementary Information

**Additional file 1.**

**Additional file 2.**

## Data Availability

All data generated or analyzed during this study are included in this published article (and its supplementary information files).

## References

[1] Saidi RF, Kazemaini SM, Malekzadeh R (2018). Current challenges of liver transplantation in Iran. Middle East J Digest Dis.

[2] Manning MW, Kumar PA, Maheshwari K, Arora H (2020). Post-reperfusion syndrome in liver transplantation—an overview. J Cardiothorac Vasc Anesth.

[3] Zhang L, Tian M, Xue F, Zhu Z (2018). Diagnosis, incidence, predictors and management of postreperfusion syndrome in pediatric deceased donor liver transplantation: a single-center study. Ann Transplant.

[4] Kork F, Rimek A, Andert A, Becker NJ, Heidenhain C, Neumann UP (2018). Visual quality assessment of the liver graft by the transplanting surgeon predicts postreperfusion syndrome after liver transplantation: a retrospective cohort study. BMC Anesthesiol.

[5] Fakhar N, Khamneh AC, Najafi A, Sharifi A, Hyder Z, Salimi J (2020). Impact of reperfusion with blood venting on liver transplantation outcomes; a prospective case-control study. Gastroenterol Hepatol Bed Bench.

[6] Siniscalchi A, Gamberini L, Laici C, Bardi T, Ercolani G, Lorenzini L (2016). Post reperfusion syndrome during liver transplantation: From pathophysiology to therapy and preventive strategies. World J Gastroenterol.

[7] Jeong S-M (2015). Postreperfusion syndrome during liver transplantation. Korean J Anesthesiol.

[8] Aggarwal S (1987). Postreperfusion syndrome: cardiovascular collapse following hepatic reperfusion during liver transplantation. Transplant Proc.

[9] Aydınlı B, Karadeniz Ü, Demir A, Güçlü ÇY, Kazancı D, Koçulu R (2016). Postperfusion syndrome in cadaveric liver transplantations: A retrospective study. Turkish J Anaesthesiol Reanimation.

[10] Zhang L, Tian M, Sun L, Zhu Z (2017). Association between flushed fluid potassium concentration and severe postreperfusion syndrome in deceased donor liver transplantation. Med Sci Monit.

[11] Tokodai K, Lannsjö C, Kjaernet F, Romano A, Januszkiewicz A, Ericzon BG (2020). Association of post-reperfusion syndrome and ischemia-reperfusion injury with acute kidney injury after liver transplantation. Acta Anaesthesiol Scand.

[12] Lee J, Yoo Y-J, Lee J-M, Park YJ, Ryu HG (2016). Sevoflurane versus Desflurane on the incidence of Postreperfusion syndrome during living donor liver transplantation: a randomized controlled trial. Transplantation..

[13] DiNorcia J, Lee MK, Harlander-Locke MP, Xia V, Kaldas FM, Zarrinpar A (2015). Damage control as a strategy to manage postreperfusion hemodynamic instability and coagulopathy in liver transplant. JAMA Surg.

[14] Patrono D, Romagnoli R. Postreperfusion syndrome, hyperkalemia and machine perfusion in liver transplantation. Transl Gastroenterol Hepatol. 2019;4:68. 10.21037/tgh.2019.08.12 PMC6789296 31620650

[15] Khosravi M, Sattari H, Ghaffaripour S, Lahssaee M, Salahi H, Sahmeddini M (2010). Post-reperfusion syndrome and outcome variables after orthotopic liver transplantation. Int J Organ Transplant Med.

[16] Siniscalchi A, Gamberini L, Bardi T, Laici C, Ravaioli M, Reggiani MLB (2017). Post-reperfusion syndrome during orthotopic liver transplantation, which definition best predicts postoperative graft failure and recipient mortality?. J Crit Care.

[17] Kalisvaart M, de Haan JE, Hesselink DA, Polak WG, Hansen BE, JN IJ (2017). The postreperfusion syndrome is associated with acute kidney injury following donation after brain death liver transplantation. Transpl Int.

[18] Droc G, Scarlatescu E, Tomescu D, Ungureanu D, Fota R, Cristea A (2013). Correlations of postreperfusion syndrome in liver transplant recipients. Am J Transplant.

[19] Peng Y, Qi X, Guo X. Child-Pugh Versus MELD Score for the Assessment of Prognosis in Liver Cirrhosis: A Systematic Review and Meta-Analysis of Observational Studies. Medicine (Baltimore). 2016;95(8):e2877. 10.1097/MD.0000000000002877 PMC4779019 26937922

[20] Chung IS, Kim HY, Shin YH, Ko JS, Gwak MS, Sim WS (2012). Incidence and predictors of post-reperfusion syndrome in living donor liver transplantation. Clin Transpl.

[21] Zalunardo MP, Schläpfer M, Beck-Schimmer B, Seifert B, Spahn DR, Bettex D (2015). Impact of cytokine release on ventricular function after hepatic reperfusion: a prospective observational echocardiographic study with tissue Doppler imaging. BMC Anesthesiol.

[22] Brezeanu L, Evans M, Milan Z, Milan Z, Ch G (2021). Anesthesia for liver transplantation. Anesthesia for Hepatico-Pancreatic-Biliary Surgery and Transplantation.

[23] Malek-Hosseini SA, Jafarian A, Nikeghbalian S, Poustchi H, Lankarani KB, Toosi MN (2018). Liver transplantation status in Iran: a multi-center report on the main transplant indicators and survival rates. Arch Iran Med.

[24] Malekzadeh MM, Vahedi H, Gohari K, Mehdipour P, Sepanlou SG, Ebrahimi Daryani N (2016). Emerging Epidemic of Inflammatory Bowel Disease in a Middle Income Country: A Nation-wide Study from Iran. Arch Iran Med..

[25] Hosseini Ahangar B, Manheouchri R, Rezaei B, Bahadori M, Ebrahimi A, Krasniq R (2020). Significant burden of nonalcoholic fatty liver disease with advanced fibrosis in iranian population: a cross-sectional analysis. Acta Med Iran..

[26] Alukal JJ, John S, Thuluvath PJ (2020). Hyponatremia in Cirrhosis: An Update. Official journal of the American College of Gastroenterology|. ACG..

[27] Attar B (2019). Approach to hyponatremia in cirrhosis. Clin Liver Dis.

[28] Zhou J, Zhang X, Lyu L, Ma X, Miao G, Chu H (2021). Modifiable risk factors of acute kidney injury after liver transplantation: a systematic review and meta-analysis. BMC Nephrol.

[29] Jung JW, Hwang S, Namgoong JM, Yoon SY, Park CS, Park YH (2012). Incidence and management of postoperative abdominal bleeding after liver transplantation. Transplant Proc.

