# Correction to: Risk factors of the post-reperfusion syndrome during orthotopic liver transplantation: a clinical observational study

**DOI:** 10.1186/s12871-022-01645-1

**Published:** 2022-04-11

**Authors:** Mohammad Ali Sahmeddini, Samaneh Ghazanfar Tehran, Mohammad Bagher Khosravi, Mohammad Hossein Eghbal, Naeimehossadat Asmarian, Fatemeh Khalili, Pooya Vatankhah, Somayeh Izadi

**Affiliations:** 1 Anesthesiology and Critical Care Research Center, Shiraz University of Medical Sciences, Shiraz, Iran; 2 Anesthesiology Research Center, Department of Anesthesiology, Alzahra Hospital, Guilan University of Medical Sciences, Rasht, Iran; 3 Shiraz Transplant Center, Abu-Alisina Hospital, Shiraz University of Medical Sciences, Shiraz, Iran

**Correction to: BMC Anesthesiol 22, 89 (2022)**

**https://doi.org/10.1186/s12871-022-01635-3**

Following publication of the original article [1], the authors identified an error in the author name of Naeimehossadat Asmarian.

The incorrect author name is: Naiemehossadat Asmarian

The correct author name is: Naeimehossadat Asmarian

The author group has been updated above and the original article [1] has been corrected.
